# Understanding the Anti-Obesity Potential of an Avocado Oil-Rich Cheese through an In Vitro Co-Culture Intestine Cell Model

**DOI:** 10.3390/molecules28155923

**Published:** 2023-08-07

**Authors:** Manuela Machado, Eduardo M. Costa, Sara Silva, Luís M. Rodriguez-Alcalá, Ana Maria Gomes, Manuela Pintado

**Affiliations:** CBQF Centro de Biotecnologia e Química Fina-Laboratório Associado, Escola Superior de Biotecnologia, Universidade Católica Portuguesa, Rua Diogo Botelho 1327, 4169-005 Porto, Portugal

**Keywords:** lipid digestion, cell-based assays, permeability, anti-inflammatory potential

## Abstract

Nowadays, with consumers’ requirements shifting towards more natural solutions and the advent of nutraceutical-based approaches, new alternatives for obesity management are being developed. This work aimed to show, for the first time, the potential of avocado oil-fortified cheese as a viable foodstuff for obesity management through complex in vitro cellular models. The results showed that oleic and palmitic acids’ permeability through the Caco-2/HT29-MTX membrane peaked at the 2h mark, with the highest apparent permeability being registered for oleic acid (0.14 cm/s). Additionally, the permeated compounds were capable of modulating the metabolism of adipocytes present in the basal compartment, significantly reducing adipokine (leptin) and cytokine (MPC-1, IL-10, and TNF-α) production. The permeates (containing 3.30 µg/mL of palmitic acid and 2.16 µg/mL of oleic acid) also presented an overall anti-inflammatory activity upon Raw 264.7 macrophages, reducing IL-6 and TNF-α secretion. Despite in vivo assays being required, the data showed the potential of a functional dairy product as a valid food matrix to aid in obesity management.

## 1. Introduction

Nowadays, the World Health Organization (WHO) considers obesity not only a disease but also a public health pandemic. It is prevalent worldwide, with 1.9 billion adults and 38.2 million children estimated to be overweight or obese. Predictions have shown that, by 2030, 50% of the world’s population could be obese [1]. Furthermore, obesity is considered a gateway pathology for other diseases as it ushers many other health-related complications such as systemic low-grade inflammation, metabolic disorders, hypertension, degenerative diseases, type 2 diabetes, cancer, and cardiovascular diseases and increases their impact on the organism [2]. Exacerbating this problem is the fact that most of the common strategies to prevent obesity are ineffective in the long term, and alternatives are urgently needed [3].

In recent years, there have been increasing efforts to find and apply alternative ways to prevent obesity as a means to decrease its impact and the costs of its metabolic consequences [4]. In this way, functional foods and nutraceuticals have emerged as alternative strategies capable of complementing classical therapeutic approaches. One of the most interesting options for this approach is the use of dairy matrices as carriers since they are highly consumed food products and already present favorable organoleptic characteristics and inherent potential health benefits [5,6]. This potential has been demonstrated using yogurt matrices [7], and of the studied options in terms of the binomial lipid source/carrier combination, avocado oil (AO)-fortified fresh cheese supplemented with probiotic microorganisms has been shown to be a suitable functional solution. Avocado oil is mainly composed of monounsaturated fatty acids (MUFAs), which have an important anti-inflammatory effect and can be used in the management of several health conditions such as obesity, diabetes, and fatty liver disease [8]. The positive impact of MUFAs in the obesity context is associated with the increased secretion of adiponectin (an adipokine responsible for regulating glucose levels and fatty acid breakdown) and reduced plasmatic pro-inflammatory cytokine levels, such as TNF-α, IL-6, IL-18, MCP-1, and INF-γ [9,10,11].

Among the possible solutions to gain further insights into the potential of the developed food prototypes, 2D co-culture models stood out.

These are practical and reproducible solutions that facilitate the evaluation of cellular and mechanistic events occurring at the intestinal interface and allow for the study of bioactive food compounds’ effects at the intestinal level [12,13,14]. While these models do not possess the detail or sensibility of ex vivo tissue or in vivo animal models, they bypass the ethical concerns, biological differences, and biopsies of human donors. Additionally, these models are extremely flexible as they may be used to evaluate compound permeability and, depending on the cells seeded in the basolateral compartment, may be used to study various conditions, ranging from inflammation to obesity, as they allow for the formation of cellular microenvironments and crosstalk between the simulated intestine and the seeded cells. While not perfect, as these models lack, for example, the complex multicellular interactions of in vivo models and cell diversity, the Caco-2/HT29-MTX co-culture model has long been accepted as a reliable model for various in vitro gut-related studies. Caco-2 and HT29-MTX cells represent the two major epithelial cell types, expressing carriers and receptors for nutrients and macromolecules in the same monolayer. The main advantage of using HT29-MTX cells is that they produce mucus, which better mimics in vivo conditions [15].

Considering all of the above, the aim of this work was to use co-culture models to evaluate the anti-obesity potential of functional probiotic cheese fortified with AO. To this end, pre-digested cheese samples were placed in the apical compartment of a Caco-2/HT29-MTX co-culture model and three different conditions were evaluated: (i) the intestinal permeability of pre-digested samples; (ii) the permeated compound’s capacity to modulate adipocyte metabolism (adipokine and cytokine production); and (iii) the permeated fatty acid macrophages’ immunomodulation as obesity is related to an inflammatory process.

## 2. Results and Discussion

### 2.1. Avocado-Rich Cheese Fatty Acid Profile

The fatty acid profile of the AO-rich fresh probiotic cheese was mainly composed of oleic acid (C18:1 c9; 55.35%), followed by palmitic (C16:0; 18.55%) and linoleic (C18:2 c9c12; 11.55%) acids (Table 1).

While the fatty acid profile of cheese digested under simulated GIT conditions showed a significant (*p* < 0.05) reduction of ca. 25% in the total amount of fatty acids, this was not reflected equally among individual fatty acids. No statistically significant differences were observed for palmitic and linoleic acids, whereas, in the case of oleic acid, only a 75% recovery in the intestinal phase was reported after passage through simulated GIT conditions.

### 2.2. Fatty Acid Permeability

Oleic and palmitic acids were the main fatty acids found in the pre-digested cheese samples, and according to the literature, they represent the two most abundant free fatty acids in the human diet [16].

**Figure 1:**
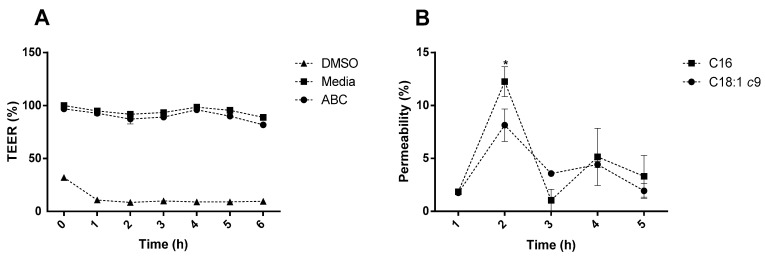
(**A**) Membrane stability as a percentage of the initial TEER value. (**B**) Oleic acid and palmitic acid permeability as a percentage of the initial compound’s concentration. * Represents the statistically significant differences (*p* < 0.05) found between sampling points. ABC—avocado cheese.

The presence of the digested cheese samples in the apical compartment slightly decreased the membrane TEER value compared to the control (Figure 1A). These results are in accordance with the literature, as some studies previously demonstrated a decrease in TEER (%) in Caco-2 monolayers and Caco-2/HT29-MTX co-cultures in the presence of oleic and palmitic acids [17,18]. As TEER measures the electrical resistance of a barrier tissue model, it is used as a quantitative parameter to evaluate the integrity of a cell layer through its ionic conductance. A reduction in TEER may be an indicator of a loosening of the membrane integrity. Because of their hydrophobic nature, free fatty acids, such as those being targeted in this study (palmitic and oleic acids), can affect lipid matrices and the lipid bilayer of the cell membrane due to their increased paracellular absorption, leading to increased fluidity [18,19]. Furthermore, the presence of oleic acid has also been related to changes in the cell phenotype, acting as a major supplier for the full reconversion of cancer cells into healthy intestinal cells [15].

Regarding the fatty acid permeability data, the results showed that low percentages of oleic (19.8%) and palmitic (23.5%) acids were found in the basolateral compartment of the cellular system (Table 2).

This is also reflected in the low apparent permeability registered for the target FAs, as a permeability of 0.14 cm/s and 0.030 cm/s for oleic acid and palmitic acid, respectively, was observed. Considering only the oleic acid permeability percentage obtained (Figure 1B), no significant differences (*p* ˃ 0.05) were observed between any sampling points, except when the highest absorption percentage was reached after 2 h. On the other hand, for palmitic acid, significant differences (*p* < 0.05) were observed at the 2 h timepoint, at which the highest absorption percentage of ca. 12% was also registered. Due to experimental drawbacks (the area under GC quantification limit), it was not possible to quantify either of the target fatty acids at the final sampling point at 6 h.

Taking into account the initial compound concentration, it was found that a substantial percentage of both oleic acid (ca. 41%) and palmitic acid (ca. 50%) was retained in the Caco-2/HT29-MTX membranes. This can be related to fatty acid absorption mechanisms, as they generally, enter the apical side of enterocytes by passive diffusion or are facilitated by the presence of transporters (among which the fatty acid translocase/cluster of differentiation 36 (FAT/CD36) plays an important role). They are then re-esterified, sequentially, inside the endoplasmic reticulum and specific binding proteins carry the fatty acids and their monoglycerides to the intracellular site, where they will be used for triglyceride biosynthesis. The two major fatty acid-binding proteins found in enterocytes are the liver fatty acid binding protein (FABP) and intestinal FABP [20,21].

### 2.3. Effect on Adipocyte Metabolism

To better mimic an in vivo scenario, the effect of the cheese sample’s permeated oleic and palmitic acids upon 3T3-L1 adipocytes was evaluated using a transwell system containing a differentiated Caco-2/HT29-MTX monolayer to simulate intestinal absorption (for 6 h), followed by a subsequent adipocyte exposure to the permeated compounds. As can be seen in Table 3, the total content of oleic and palmitic acids in the basolateral chamber was 2.45 ± 0.04 µg/mL and 1.12 ± 0.05 µg/mL, respectively. Moreover, considerable amounts of oleic acid (19.22 ± 11.30 µg/mL) and palmitic acid (26.68 ± 6.88 µg/mL) were quantified in the 3T3-L1 cells. This means that part of the fatty acids capable of permeating the membrane were found within the 3T3-L1 adipocytes. Additionally, in the Caco-2/HT29-MTX membranes, high amounts of oleic (75.25 ± 24.90 µg/mL) and palmitic acids (12.64 ± 1.33 µg/mL) were also quantified. This result may be explained by a previous work by Berger and Géloen [22] which showed that concentrations between 20 to 40 µM of oleic acid promoted an increase in triglycerides’ uptake by enterocytes in Caco-2/HT29-MTX membranes when in the presence of adipocytes [22].

**Figure 2:**
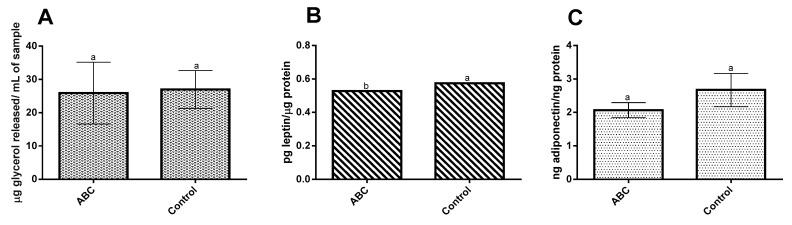

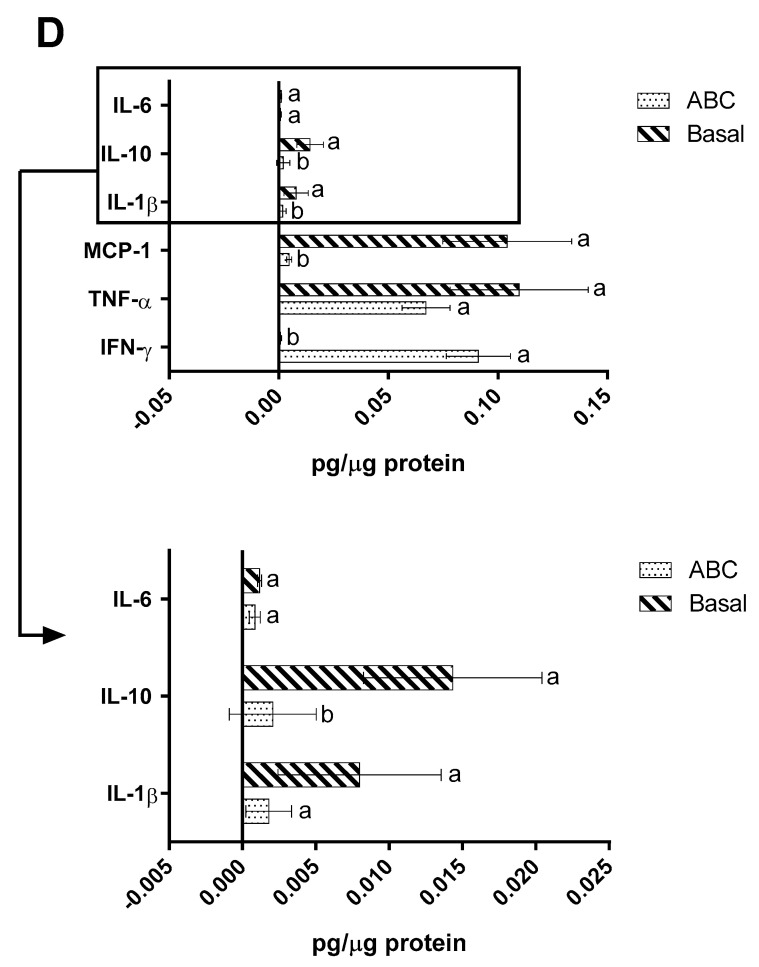
Effect of fatty acids from pre-digested fresh avocado oil bigel-fortified cheese (ABC) on adipolysis and adipokine secretion and cytokine secretion in the 3T3-L1 complex co-culture model basolateral side by the Caco-2/HT29-MTX membrane. (**A**) Adipolysis; (**B**) leptin secretion; (**C**) adiponectin secretion; and (**D**) cytokine secretion. Different letters indicate significant differences between conditions as determined by a one-way ANOVA test (*p* < 0.05). ABC—avocado cheese.

Concerning adipolysis (Figure 2), no significant differences (*p* ˃ 0.05) were observed between the controls and cells treated with the digested cheese samples (Figure 2A), as the presence of 3T3-L1 lipid accumulation in the cheese samples only slightly increased (4.1%). When concerning the adipokine data, the results obtained regarding leptin, an adipokine responsible for the regulation of fat storage and which exerts mostly pro-inflammatory and immune-potentiating effects, showed that the presence of these fatty acids significantly (*p* < 0.05) reduced (8.77%) leptin secretion (Figure 2B). These results were opposite to those previously reported by another author in which the presence of oleic and palmitic acids (in concentrations up to 0.25 mM) increased leptin secretion [23]. A possible explanation for this discrepancy may be related to the concentrations used in each study: the oleic (39.6 µM) and palmitic (95.5 µM) acid concentrations used in our work are 0.15 and 0.38 times lower than those of the published assay. On the other hand, no significant differences (*p* ˃ 0.05) were observed in adiponectin secretion (Figure 2C). Despite this, a ca. 29% decrease was observed when compared to the basal activity. Considering that adiponectin exerts an anti-inflammatory and anti-diabetic effect, these results may represent an unfavorable result. However, the literature on these fatty acids’ activity is not consensual, as previous works have shown that both palmitic acid (at concentrations between 10 and 100 µM) and oleic acid (at 100 µM and under 10 µM) lead to increases in adiponectin secretion [24,25,26], and others have shown that oleic acid and palmitic acid (up to 0.5 mM) reduced adiponectin secretion [23]. Considering that the concentrations used in this work fall within the intervals depicted in the literature for both the promotion and inhibition of this adipokine secretion, no objective conclusions could be reached.

Inflammation in the adipose tissue is an important factor associated with the development of obesity-related diseases such as type 2 diabetes and metabolic syndrome [16]. Previous works have shown that the presence of dietary fatty acids (such as palmitic and oleic acid) can modulate the inflammatory status of 3T3-L1 adipocytes [27]. As can be seen in Figure 2D, significant differences (*p* < 0.05) were observed for MCP-1, IL-10, IL-1β, and INF-γ secretion.

MCP-1 is responsible for macrophage recruitment and its levels have been shown to be increased in high-fat diets [16]. In this work, the secretion of MCP-1 was significantly (*p* < 0.05) reduced (ca. 96%) in comparison to the basal production. Previously published studies showed that the presence of palmitic acid, at concentrations between 5 and 25 mM, increased the secretion of MCP-1. However, these values are vastly superior to the 95.5 µM quantified in this work and which presented an inhibitory activity upon MCP-1 secretion [24,25,28]. On the other hand, small amounts of oleic acid (1–10 µM) have been previously shown to block MCP-1 secretion, a behavior which was also here observed despite the higher oleic acid concentrations registered (39.6 µM). This inhibitory effect could be related to the MUFAs’ capacity to inhibit the NF-kB signaling pathway [25]. A significant (*p* < 0.05) reduction was also observed for IL-10 secretion, with the IL-10 concentration in 3T3-L1 cells treated with digested cheese samples being 85% lower than that registered for the control. According to the data available in the literature, this reduction can be explained by the presence of palmitic acid. A study published by Bradley et al. in 2008 showed that palmitic acid, at concentrations similar to those used in this work (50–500 µM), reduced IL-10 secretion. In the same study, the authors demonstrated that the presence of oleic acid did not exert any effect on IL-10 secretion, a result contrary to the inhibition observed here at 39.6 µM (although the work was carried out above the range of concentrations reported in that work) [29]. Regarding IL-1β, a 78% reduction was observed compared to the basal activity. These results are in line with previous in vivo studies which showed that diets rich in MUFAs (oleic acid) significantly reduced IL-1β secretion [30,31]. In the case of IFN-γ, a significant (*p* < 0.05) increase (Ca 99%) was observed. Previous animal studies with mice fed diets rich in MUFAs showed that the presence of MUFAs did not exert significant effects on IFN-γ levels [32]. Lastly, no significant differences (*p* ˃ 0.05) were observed for IL-6 and TNF-α secretion. The same result was previously obtained by Bradley et al. (2008) for TNF-α in 3T3-L1 cells treated with oleic acid at concentrations slightly superior (39.6 µM vs. 50 to 500 µM) to the ones registered in this work [29]. Another study showed that 3T3-L1 adipocytes treated with 20, 100, and 500 µM of oleic acid did not show significant changes in TNF-α secretion. In the same study, only the higher concentrations tested were responsible for an increase in IL-6 secretion [26]. Moreover, a study with human adipocytes demonstrated that oleic acid (5–10 µM) did not exert an upregulation of IL-6 and TNF-α secretion [33].

### 2.4. Immunomodulation Model

The immunomodulatory capacity was evaluated in Raw 264.7 cells exposed to the permeated fatty acids from the digested cheese samples. In this case, 2.16 ± 0.21 µg/mL of oleic acid and 3.30 ± 0.15 µg/mL of palmitic acid were quantified in the cell supernatants (corresponding to the basolateral chamber) (Table 4). Additionally, the results also showed that 11.12 ± 1.24 µg/mL of oleic acid and 9.26 ± 0.15 µg/mL of palmitic acid were internalized by Raw 264.7 cells and that 35.9% and 18.7% of the initial amounts of oleic and palmitic acids, respectively, were retained by the Caco-2/HT29-MTX membrane.

**Figure 3:**
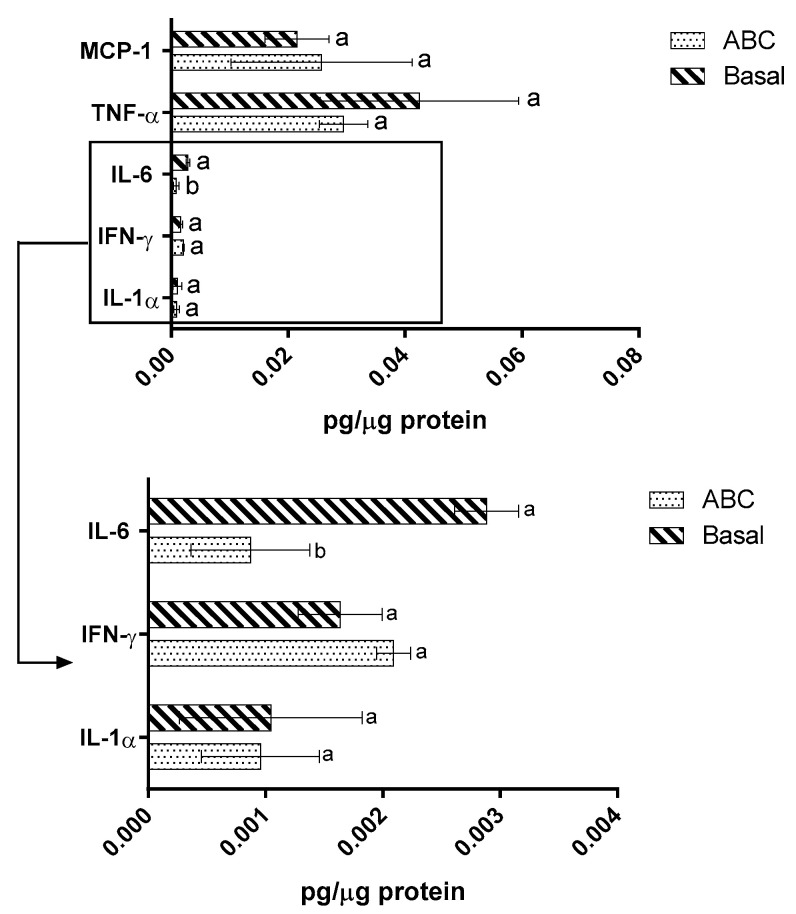
Cytokine production in the Raw 264.7 complex co-culture model basolateral side by the Caco-2/HT29-MTX membrane. Different letters represent the statistically significant differences (*p* < 0.05) found between conditions for each cytokine. ABC—avocado cheese.

When considering the impact of the samples upon the cytokine secretion of the Raw 264.7 cells, the presence of these fatty acids led to a significant (*p* < 0.05) decrease (ca. 60%) in IL-6 secretion (Figure 3). Previous studies showed that oleic acid was responsible for a reduction in IL-6 secretion [10,11,34], with the possible mechanism behind this reduction being related to the MUFAs’ ability to inhibit the NF-kB and the NOD-like receptor family pyrin domain activation and inhibit the PPAR [27].

No significant (*p* ˃ 0.05) differences were observed for MCP-1, TNF-α, INF-γ, and IL-1α. Previous studies have shown that palmitic acid (at concentrations 400–500 µM) was responsible for an increase in pro-inflammatory cytokine secretion in Raw 264.7 cells through the activation of toll-like receptor 4 (TLR4)-dependent signaling pathways and activation of both MAPK signaling and the NF-kB pathway [35,36]. On the other hand, oleic acid (200–750 µM) reduced pro-inflammatory cytokines and pro-inflammatory lipid mediators secretion, with this effect being dependent on the concentration and exposure time [37,38]. This may be related to the inhibition of NF-kB activation as mentioned above and the suppression of the TLR4 signaling pathway [36]. Considering that, in this work, palmitic acid was found at 84.2 µM and oleic at 116.8 µM, values well below the concentrations which showed activity in previous works, it is possible that the lack of differences may be attributed to this factor.

## 3. Materials and Methods

### 3.1. Avocado Bigel and Cheese Production

Avocado bigel was produced as described by Machado et al. (2023) [39] and the cheese was produced according to Machado et al. (2023) [40]. In brief, commercially available skimmed milk powder was dissolved in water (13% *w*/*v*). Following this, avocado oil bigel (3% *w*/*w*) was added to the suspension and the mixture was pasteurized for 2 min at 90 °C using a Bimby TM6 (Vorwerk Thermonix, Wuppertal, Germany). Samples were then cooled to 37 °C and the probiotic strains (10% *w*/*v*) were added. Cheese coagulation was then started through the addition of a lactic acid solution (20% *v*/*v*), and the samples were mixed for 2 min at 37 °C. After 10 min, the curd was cut and separated using a hanging cloth and left to drain for 5 min. Cheeses were placed in a plastic container and stored at 4 °C.

### 3.2. Fatty Acid Profile

The fatty acid profile was evaluated by gas chromatography after transesterification according to the method previously described by Machado et al. (2022) [41]. Fatty acid methyl esters were analyzed in a gas chromatograph Agilent 8860 (Agilent, Santa Clara, CA, USA) equipped with a flame ionization detector and a BPX70 capillary column (60 m × 0.25 mm × 0.25 μm; SGE Europe Ltd., Courtaboeuf, France). Analysis conditions were as follows: injector (split 25:1; injection volume 1 µL), injector and detector temperatures were 250 °C and 275 °C, respectively, and hydrogen was used as a carrier gas at a flow rate of 1 mL/min. The oven temperature was initially at 60 °C and then increased to a final temperature of 225 °C. Supelco 37 certified reference material was used for the identification and quantification of fatty acids. Each sample was analyzed in triplicate.

### 3.3. In Vitro Simulation of the GIT

The in vitro gastrointestinal tract (GIT) simulation was performed according to the INFOGEST method [42], in triplicate, using 5 g of sample. After the intestinal phase, 1 mL aliquots were collected to determine the fatty acid profile (as described above) and the remaining sample was aliquoted and stored at −30 °C for further in vitro assays.

### 3.4. Cellular Models

#### 3.4.1. Cell Lines

Five different cell lines were used in the current work: Human Caucasian colon carcinoma epithelial cells (Caco-2, ECACC 86010202) and HT29-MTX E12 (ECACC 12040401) were acquired from the European Collection of Authenticated Cell Cultures mouse macrophages Raw 264.7 (ATCC TIB-71), and mouse pre-adipocytes 3T3-L1 (ATCC CL-173) were acquired from American Type Culture Collection (Manassas, VA, USA). Human cell lines and mouse macrophages were cultured using DMEM (Gibco, Thermo Scientific, Waltham, MA, USA) supplemented with 10% (*v*/*v*) FBS from Biowest (Nuaillé, France) and 1% (*v*/*v*) of Penicillin–Streptomycin–Fungizone (Lonza, Switzerland). For Caco-2 and HT29-MTX, media was also supplemented with 1% (*v*/*v*) non-essential amino acids (Gibco, Thermo Scientific, Waltham, MA, USA). Pre-adipocytes were cultured in DMEM with 10% (*v*/*v*) of iron-fortified CBS (ATCC, Manassas, VA, USA) and 1% (*v*/*v*) of Penicillin–Streptomycin–Fungizone. All cell lines were incubated at 37 °C under a humidified atmosphere comprised of 5% CO_2_ and 95% air.

#### 3.4.2. Co-Culture Models

Co-culture models were performed through adaptation of the methods previously described by Antunes et al. (2013) [43]. Briefly, Caco-2/HT29-MTX co-cultures were seeded on the apical chamber of a 12-well Transwell (Corning, New York, NY, USA) plate in a 90:10 proportion, respectively, to reach a monolayer with a final density of 1 × 10^5^ cells/cm^2^ in each insert; these were then maintained for 21 days until assaying.

#### 3.4.3. Transepithelial Electrical Resistance (TEER) Measurements

In all assays, the membrane integrity of the different models was evaluated through the measurement of transepithelial electrical resistance (TEER) using a Millicell ERS-2 Voltohmmeter (Merck, Germany). It should be noted that only the monolayers with TEER values between 150 and 250 Ω/cm^2^ were selected for permeability experiments.

#### 3.4.4. Fatty Acid Permeability Assay

Permeability assays were performed through adaptation of the methods previously described by Antunes et al. (2013) [43]. Briefly, the 21 days differentiated Caco-2/HT29-MTX co-culture media in the apical chamber was replaced either with plain media (basal control), media with cheese digested sample (200 µg/mL of fatty acids), or with DMSO at 10% (*v*/*v*) (stress control), and the plate was re-incubated for 6 h. Every hour, the cell monolayer’s integrity was measured, a 0.5 mL aliquot was collected, and the volume removed was replaced with the medium. After 6 h, the media in both compartments was collected. The cell membranes were also collected using 0.1 M of sodium hydroxide and stored at −80 °C until analysis. In all sampling points, the fatty acid profile was evaluated as described above. Each sample was evaluated in duplicate. The apparent permeability was calculated according to the following equation:

(1) $$Papp\left(cm/s\right)=\frac{dQ}{dt(A\times C0)}$$

where dQ is the total amount of permeated fatty acid (µg/mL), A is the diffusion area (cm^2^), C0 is the initial concentration of fatty acids (µg/mL), and dt is the time of the experiment(s). The coefficient dQ/dt represents the flux of fatty acids across the monolayer.

#### 3.4.5. Modulation of Adipocyte Metabolism Using a Co-Culture Model

The adipocyte metabolism modulation was performed as subsequently described. First, pre-adipocytes were seeded (4 × 10^4^ cells/well) into 12-well plates and chemically differentiated according to the ATCC guidelines as previously described by Machado et al. (2022) [44]. After differentiation, 21 d old Caco-2/HT29-MTX co-culture inserts were transferred from the Transwell plate and placed over the adipocyte’s monolayer, after which media in the apical chamber was replaced with plain media (basal control), media with pre-digested cheese (200 µg/mL of fatty acids), or with DMSO at 10% (*v*/*v*) (stress control) and the plate was re-incubated for 6 h. Following incubation, the apical medium was collected, inserts were removed, and the plates were incubated for 18 h. Cell membranes were removed from the insert as described above. After 24 h, the supernatants and adipocytes were collected and stored at −80 °C for further analysis. The fatty acid profile of apical, basal samples, cell membranes (Caco-2/HT29-MTX), and 3T3-L1 cells was determined as described above. All assays were performed in duplicate.

The modulation of obesity metabolism upon the 3T3-L1 cells present in the basolateral compartment was evaluated upon cells’ adipolysis and adipokine secretion. Adipolysis was evaluated as previously described by Machado et al. (2022) [44]. Adipokine modulation was performed via the detection of adiponectin and leptin secretion through ELISA assays (Abcam’s mouse leptin ELISA kit and mouse adiponectin ELISA kit (Abcam, Cambridge, UK), with each sample being analyzed in triplicate. Leptin values were obtained in pg/µg of protein in the sample and adiponectin in ng/ng of protein.

Immunomodulatory effects upon 3T3-L1 adipocytes were analyzed using a 13-analyte mouse multiplex panel (LEGENDplex, Biolegend, San Diego, CA, USA) according to the manufacturer’s instructions. Results were obtained using a BD Accuri™ C6 flow cytometer (BD, Franklin Lakes, NJ, USA) gated according to the multiplex manufacturer’s instructions, and the results were expressed in pg/µg protein. Each sample was analyzed in quadruplicate.

#### 3.4.6. Raw 264.7 Co-Culture Intestinal Inflammatory Model

Raw 264.7 were seeded (8 × 10^5^ cells/well) into 12-well plates and allowed to adhere overnight. Afterward, 21 d old Caco-2/HT29-MTX co-culture inserts were placed over the adhered Raw 264.7 cells. The medium in the apical chamber was replaced with plain medium (basal control) or medium with the pre-digested cheese sample (200 µg/mL of fatty acids) and the plate was re-incubated for 6 h. After this period, the inserts were removed and the plates were incubated for 18 h. Cell membranes were removed from the insert as described above. Supernatants and Raw 264.7 cells were collected and stored at −80 °C for further analysis. The fatty acid profile of apical, basal samples, cell membranes, and Raw 264.7 cells was determined as described above. All assays were performed in duplicate.

The immunomodulatory effects upon Raw 264.7 cells present in the basolateral compartment were examined using a 13-analytes mouse multiplex panel (LEGENDplex, Biolegend, San Diego, CA, USA) according to the manufacturer’s instructions. Results were obtained using a BD Accuri™ C6 flow cytometer (BD, Franklin Lakes, NJ, USA) gated according to the multiplex manufacturer instructions and the results given in pg/µg protein. All samples were analyzed in quadruplicate.

### 3.5. Statistical Analysis

Minitab 17 (Minitab, State College, PA, USA) was used to carry out statistical analysis. All data were reported as the mean ± standard deviation. Shapiro–Wilk’s test was used to confirm the normality of data distribution. The results obtained were tested at a 0.05 significance level using a one-way ANOVA, followed by a multiple comparisons test (Tukey) to identify statistically significant differences between samples.

## 4. Conclusions

Overall, the data here presented showed that the developed dairy matrix allowed for the delivery of bioactive fatty acids in high enough quantities to be absorbed by the intestinal wall and exert effective modulation on the obesity-related target cells. In detail, oleic and palmitic acids were delivered in concentrations (50.5 µg/mL and 116.8 µg/mL) capable of being absorbed and capable of downregulating leptin, MCP-1, IL-10, IL1-β, TNF-α, and IL-6 secretion in adipocytes and IL-6 and TNF-α secretion in macrophages in the adipocyte metabolism and immunomodulation Caco-2/HT29-MTX co-culture models. Despite in vivo assays being required to validate this data in a more complex system, the results obtained showed, unequivocally and for the first time ever, the potential of a bioactive fatty acid-rich functional dairy product as a valid food to manage obesity and offered insights into the fatty acid permeation of the intestinal wall and subsequent obesity metabolism modulation.

## Figures and Tables

**Table 1:** Fatty acid profile of avocado oil bigel-fortified fresh cheese before and after simulated GIT passage.

	Before GIT	After GIT
C14	0.14 ± 0.001 ^a^	0.14 ± 0.01 ^a^
C16	3.63 ± 0.09 ^a^	3.53 ± 0.42 ^a^
C16:1 c9	0.77 ± 0.03 ^a^	0.60 ± 0.09 ^b^
C18	0.82 ± 0.01 ^b^	1.22 ± 0.09 ^a^
C18:1 c9	10.83 ± 0.31 ^a^	8.17 ± 0.94 ^b^
C18:1 c11	0.80 ± 0.02 ^a^	0.61 ± 0.08 ^b^
C18:2 c9c12	2.26 ± 0.08 ^a^	2.31 ± 0.34 ^a^
C20	0.14 ± 0.01 ^a^	0.12 ± 0.01 ^a^
C18:3 c6c9c12	0.10 ± 0.01 ^a^	0.08 ± 0.01 ^a^
C20:1	0.07 ± 0.01 ^a^	0.05 ± 0.01 ^a^
∑Fatty acids	19.57 ± 0.51 ^a^	16.83 ± 1.98 ^b^

Results are expressed in mg/g and represent the means of three independent determinations ± standard deviation. Different letters indicate significant differences as determined by a one-way ANOVA test (*p* < 0.05). C14 myristic acid; C16 palmitic acid; C16:1 c9 palmitoleic acid; C18 stearic acid; C18:1 c9 oleic acid; C18:1 c11 cis-vaccenic acid; C18: 12 c9c12 linoleic acid; C18:3 c6c9c12 γ-linolenic acid; and C20:1 cis-gondoic acid.

**Table 2:** The fatty acid content in the apical and basolateral chamber, Caco-2/HT29-MTX monolayer, and the compounds’ apparent permeability.

	Initial (µg/mL)	Apical (µg/mL)	Membrane (µg/mL)	Basolateral (µg/mL)	P_app_ (cm/s)
C16	50.5 ± 1.83 ^a^	1.24 ± 1.18 ^d^	25.40 ± 1.70 ^b^	11.89 ± 1.15 ^c^	0.03 ± 0.01
C18:1 c9	116.8 ± 8.33 ^a^	5.57 ± 0.01 ^c^	48.04 ± 2.82 ^b^	23.14 ± 6.84 ^b^	0.14 ± 0.04

The content of oleic (C18:1 c9) and palmitic acid (C16) was assessed after 6 h of exposure. Results represent the means of three independent determinations ± standard deviation. Different letters in the same row indicate significant differences as determined by a one-way ANOVA test (*p* < 0.05).

**Table 3:** The palmitic and oleic acid content in the apical and basolateral chambers, Caco-2/HT29-MTX membranes, and uptake by 3T3-L1 cells.

	6 h				24 h
	Initial	Apical	Membrane	Basolateral	Cells
C16	50.5 ± 1.83 ^b^	6.18 ± 3.71 ^b^	12.64 ± 1.33 ^b^	1.12 ± 0.05 ^b^	26.68 ± 6.88 ^a^
C18:1 c9	116.8 ± 8.33 ^a^	19.29 ± 3.71 ^a^	75.25 ± 24.90 ^a^	2.45 ± 0.04 ^a^	19.22 ± 11.30 ^a^

The content of oleic (C18:1 c9) and palmitic acid (C16) are expressed in µg/mL and represent the means of three independent determinations ± standard deviation. Different letters indicate significant differences as determined by a one-way ANOVA test (*p* < 0.05).

**Table 4:** The fatty acid content in the apical and basolateral chambers, Caco-2/HT29-MTX membranes, and uptake by Raw 264.7 cells.

	6 h				24 h
	Initial	Apical	Membrane	Basolateral	Cells
C16	50.5 ± 1.83 ^b^	10.69 ± 5.36 ^a^	9.43 ± 3.73 ^b^	3.30 ± 0.15 ^a^	11.12 ± 1.24 ^a^
C18:1 c9	116.8 ± 8.33 ^a^	10.52 ± 4.83 ^a^	41.94 ± 4.00 ^a^	2.16 ± 0.21 ^b^	9.26 ± 0.15 ^b^

The content of oleic (C18:1 c9) and palmitic acid (C16) are expressed in µg/mL and represent the means of three independent determinations ± standard deviation. Different letters indicate significant differences as determined by a one-way ANOVA test (*p* < 0.05).

## Data Availability

The data presented in this study are available on request from the corresponding author.
